# Digital reconstruction of infraslow activity in human intracranial ictal recordings using a deconvolution-based inverse filter

**DOI:** 10.1038/s41598-022-18071-5

**Published:** 2022-08-11

**Authors:** Somin Lee, Julia Henry, Andrew K. Tryba, Yasar Esengul, Peter Warnke, Shasha Wu, Wim van Drongelen

**Affiliations:** 1 Medical Scientist Training Program, The University of Chicago, Chicago, IL 60637 USA; 2 Committee on Neurobiology, The University of Chicago, Chicago, IL 60637 USA; 3 Department of Pediatrics, The University of Chicago, Chicago, IL 60637 USA; 4 Department of Neurology, The University of Chicago, Chicago, IL 60637 USA; 5 Department of Neurosurgery, The University of Chicago, Chicago, IL 60637 USA; 6 Committee On Computational Neuroscience, The University of Chicago, Chicago, IL 60637 USA

**Keywords:** Epilepsy, Signal processing

## Abstract

Infraslow activity (ISA) is a biomarker that has recently become of interest in the characterization of seizure recordings. Recent data from a small number of studies have suggested that the epileptogenic zone may be identified by the presence of ISA. Investigation of low frequency activity in clinical seizure recordings, however, has been hampered by technical limitations. EEG systems necessarily include a high-pass filter early in the measurement chain to remove large artifactual drifts that can saturate recording elements such as the amplifier. This filter, unfortunately, attenuates legitimately seizure-related low frequencies, making ISA difficult to study in clinical EEG recordings. In this study, we present a deconvolution-based digital inverse filter that allows recovery of attenuated low frequency activity in intracranial recordings of temporal lobe epilepsy patients. First, we show that the unit impulse response (UIR) of an EEG system can be characterized by differentiation of the system’s step response. As proof of method, we present several examples that show that the low frequency component of a high-pass filtered signal can be restored by deconvolution with the UIR. We then demonstrate that this method can be applied to biologically relevant signals including clinical EEG recordings obtained from seizure patients. Finally, we discuss how this method can be applied to study ISA to identify and assess the seizure onset zone.

## Introduction

Epilepsy is a prevalent neurological condition that affects over three million people in the United States^1^. A third of patients with epilepsy continue to experience seizures despite medication treatment^2^. Although many of these patients pursue surgical options, 40–70% of surgical patients continue having seizures after resective surgery^3^. Surgical intervention is thought to fail in these cases due to the incomplete removal of the culprit brain tissue, a putative and theoretical area known as the epileptogenic zone (EZ). Because the EZ is an area that can only be defined post-surgically, the seizure onset zone (SOZ) is used as a proxy for the EZ during surgical planning^4^. One possible reason for the limited efficacy of surgical interventions is that our ability to identify and delineate the SOZ is insufficient. Thus, identifying features that are unique to the SOZ/EZ has great potential for improving surgical outcomes.

Low frequency ictal activity is a biomarker that has recently become of interest in the characterization of seizure recordings. Recent data from a number of studies suggest that the SOZ may be identified by the presence of very low frequency oscillations^5–7^ (for a review, see Lee et al.^8^). Termed “infraslow activity” (ISA) or direct current (DC) shifts in the literature, this low frequency band is typically defined as activity below 0.1 or 0.5 Hz. This frequency band is much slower than the 1–70 Hz band at which clinical EEGs are typically interpreted. The study of low frequency activity in seizure recordings, however, has been hampered by technical limitations. EEG systems necessarily include a high-pass filter early in the measurement chain to remove large artifactual drifts that can saturate recording elements such as the amplifier. This filter unfortunately attenuates legitimately seizure-related low frequencies, making ISA essentially impossible to study in clinical EEG recordings. Although a few studies have utilized DC amplifiers to try to bypass this limitation^9–12^, DC amplifiers are not immune to saturation issues (Supplementary Fig. S1). Furthermore, standard clinical EEG equipment does not utilize DC amplifiers, making observing ISA directly in clinical recordings difficult. Consequently, development of a method to evaluate ISA in recordings obtained with alternating-current (AC) amplifiers is desirable.

In this study, we present a novel approach to digitally reconstruct attenuated low frequency activity in clinical EEG recordings using a deconvolution-based inverse filter. First, we show that a clinical EEG system’s unit impulse response (UIR) may be derived by differentiation of the system’s step response. We then use this UIR to deconvolve a variety of synthetic signals to demonstrate successful restoration of attenuated low frequencies. We then show that this method is stable when applied to clinical intracranial recordings obtained from temporal lobe epilepsy patients. Finally, we discuss how this method may be applied to study ISA as it relates to the identification and assessment of the SOZ.

## Results

**Figure 1:**
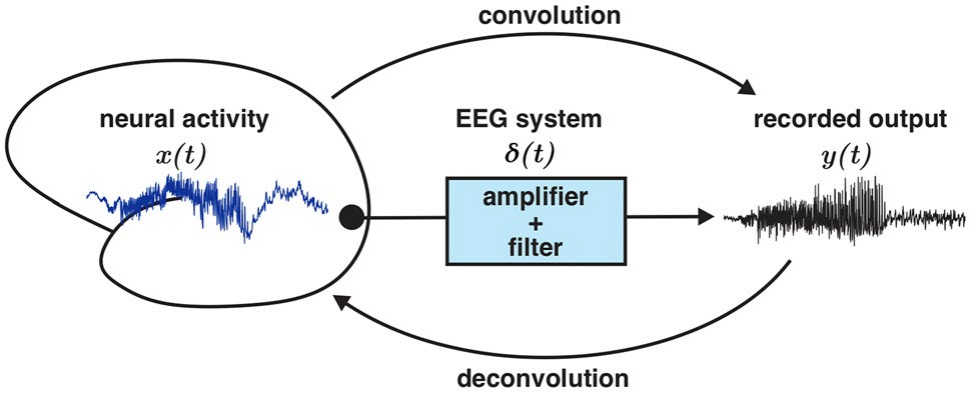
Elements of a linear time invariant (LTI) system may be described by convolution and deconvolution operations. In a LTI system, output function *y*(*t*) may be described by a convolution of the input signal *x*(*t*) with the system’s unit impulse response *δ*(*t*). Inversely, the input signal may be obtained by deconvolving *y*(*t*) with *δ*(*t*). The unit impulse response fully characterizes the LTI system in the time domain. In this schematic, the LTI system is the EEG recording machinery that includes elements such as amplifiers and high-pass filters.

In linear time invariant (LTI) systems, the effects of recording elements such as filters on signals may be mathematically described as a convolution of the input signal with the system’s characteristic unit impulse response (UIR) (Fig. 1). Inversely, the input of this system can be found by deconvolution of the output signal with the UIR (Fig. 1). If the LTI element in question is a filter, the deconvolution operation may be thought of as an “inverse filter” that allows for recovery of frequencies attenuated by the filter. While this concept is mathematically straightforward, the challenge of applying this method to real-world signals lies in the ability to accurately characterize the UIR of the recording system.

### Characterization of the unit impulse response of a clinical EEG system

**Figure 2:**
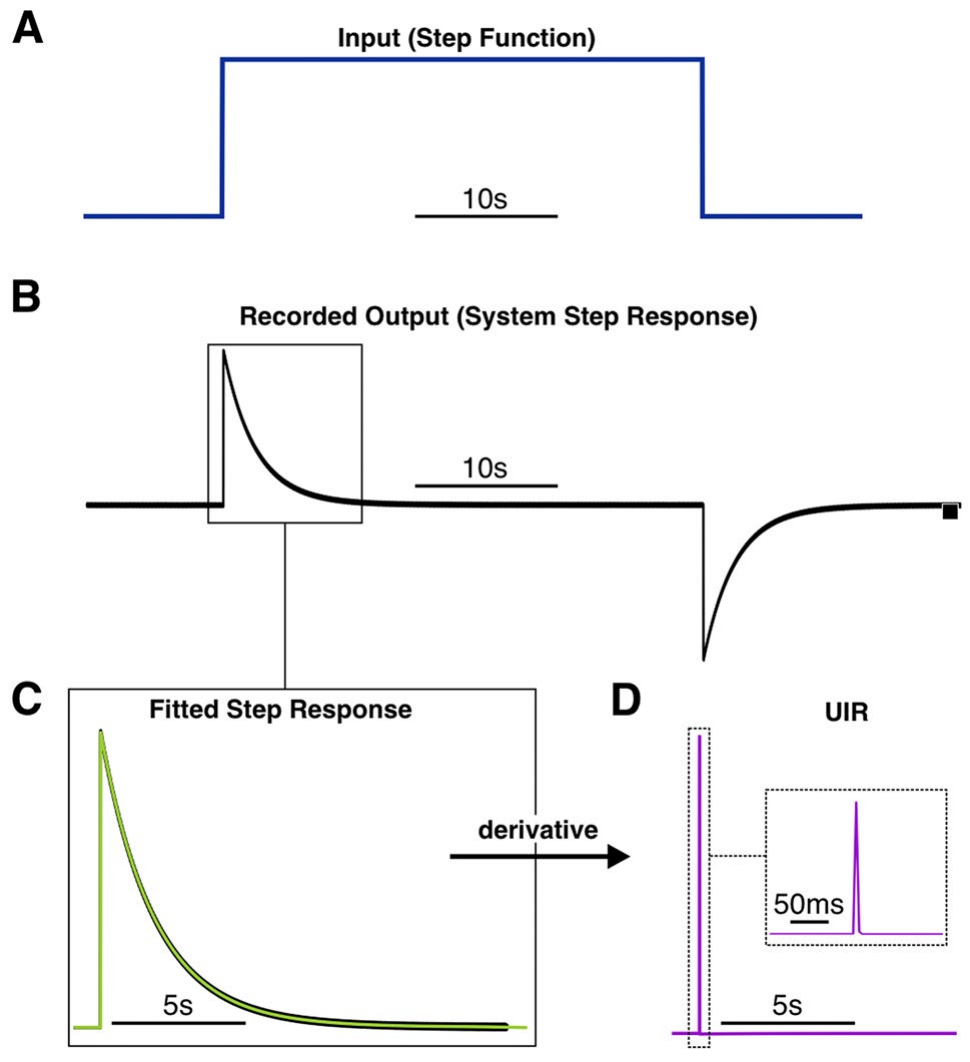
Determination of an EEG system’s unit impulse response (UIR) by measuring the system’s step response. Amplitude for all panels is in arbitrary units. A synthetically generated step function was used as the input signal into the EEG system (**A**). The measured output was the system’s step response (**B**). This step response was fitted with a 9th order polynomial (light green trace) (**C**). Taking the derivative of this polynomial function resulted in the system’s UIR (purple trace). Dotted inset shows a zoomed in view of the peak of the UIR (**D**).

**Figure 3:**
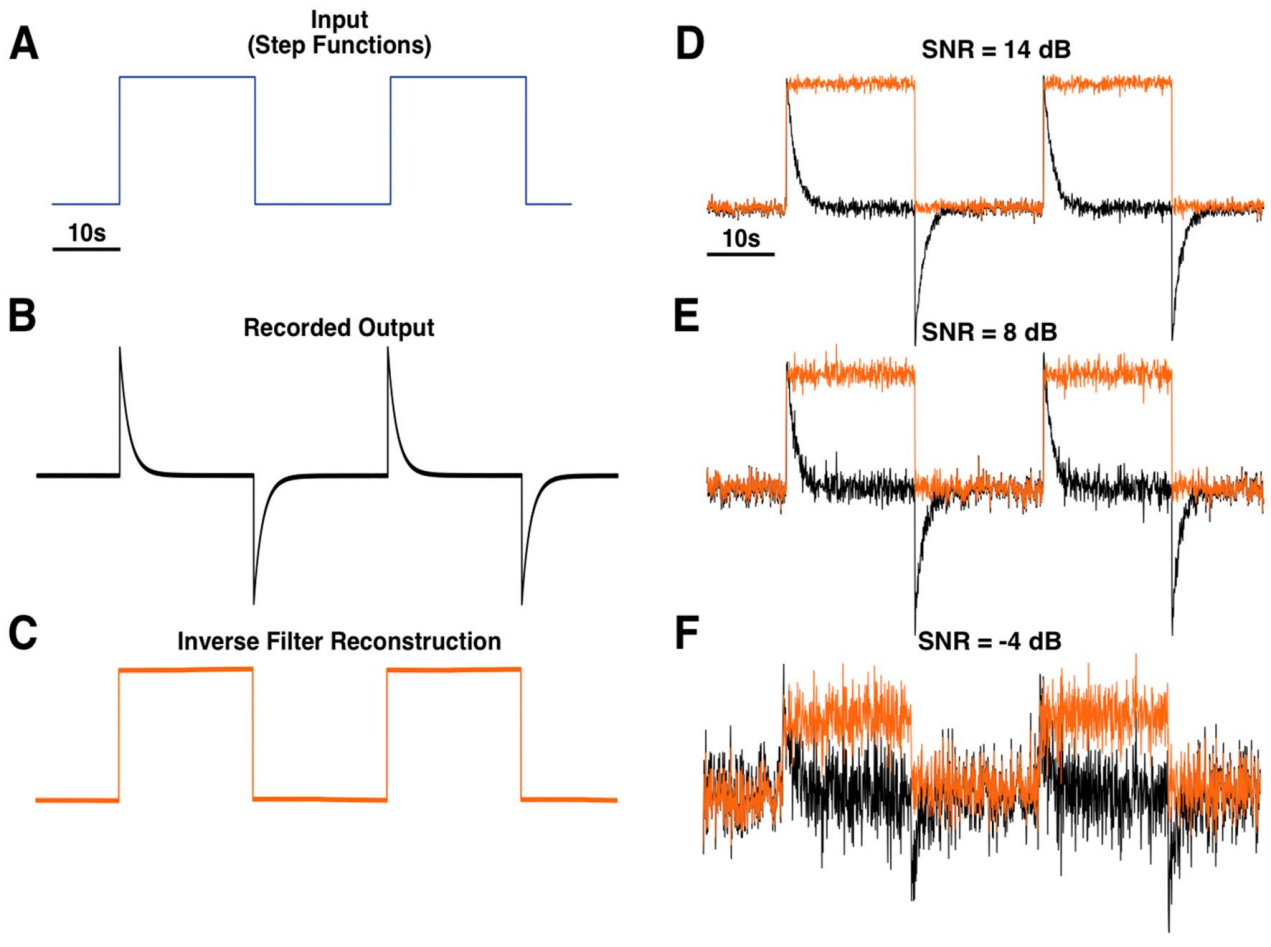
Application of the deconvolution-based inverse filter to step functions. A series of two step functions was used as a test input signal (**A**). The EEG system’s recorded output (**B**) was deconvolved by the unit impulse response (UIR) depicted in Fig. 2D, which resulted in the reconstruction of the original step function (**C**). This method was able to reconstruct the general step function shape with high fidelity even with the addition of varying levels of normally distributed random noise (**D**–**F**). In panels (**D**–**F**), the black traces show the recorded output with added noise, and the orange traces show the inverse filtered reconstructions. *SNR* signal-to-noise ratio.

Mathematically, the UIR of a system is the derivative of the system’s step response^13^. To characterize the UIR of a clinical EEG unit (Natus XLTEK Brain monitor with Connex headbox), we used a digital/analog (D/A) converter to input a synthetically generated step function (Fig. 2A) (“Methods”). The output measured by the EEG system (i.e., the system’s step response) was fitted with a 9th order polynomial (Fig. 2B,C). Taking the derivative of this polynomial approximation resulted in the putative characteristic UIR of the recording system (Fig. 2D). The accuracy of this UIR was verified by deconvolving the recorded output of a test function with known low frequency activity. The test function was a series of two step functions (Fig. 3A). Deconvolution of the recorded output of these step functions (Fig. 3B) with the UIR resulted in a reconstruction that resembled the input (Fig. 3C). Notably, the flat feature of the step functions was restored, confirming that this method is appropriate for reconstructing DC shifts. The deconvolution operation was also robust to noise, as the square shape of the reconstruction was preserved even with the addition of high levels of normally distributed random noise (Fig. 3D–F).

### Validation of deconvolution-based inverse filter using synthetic signals with known low frequency components

**Figure 4:**
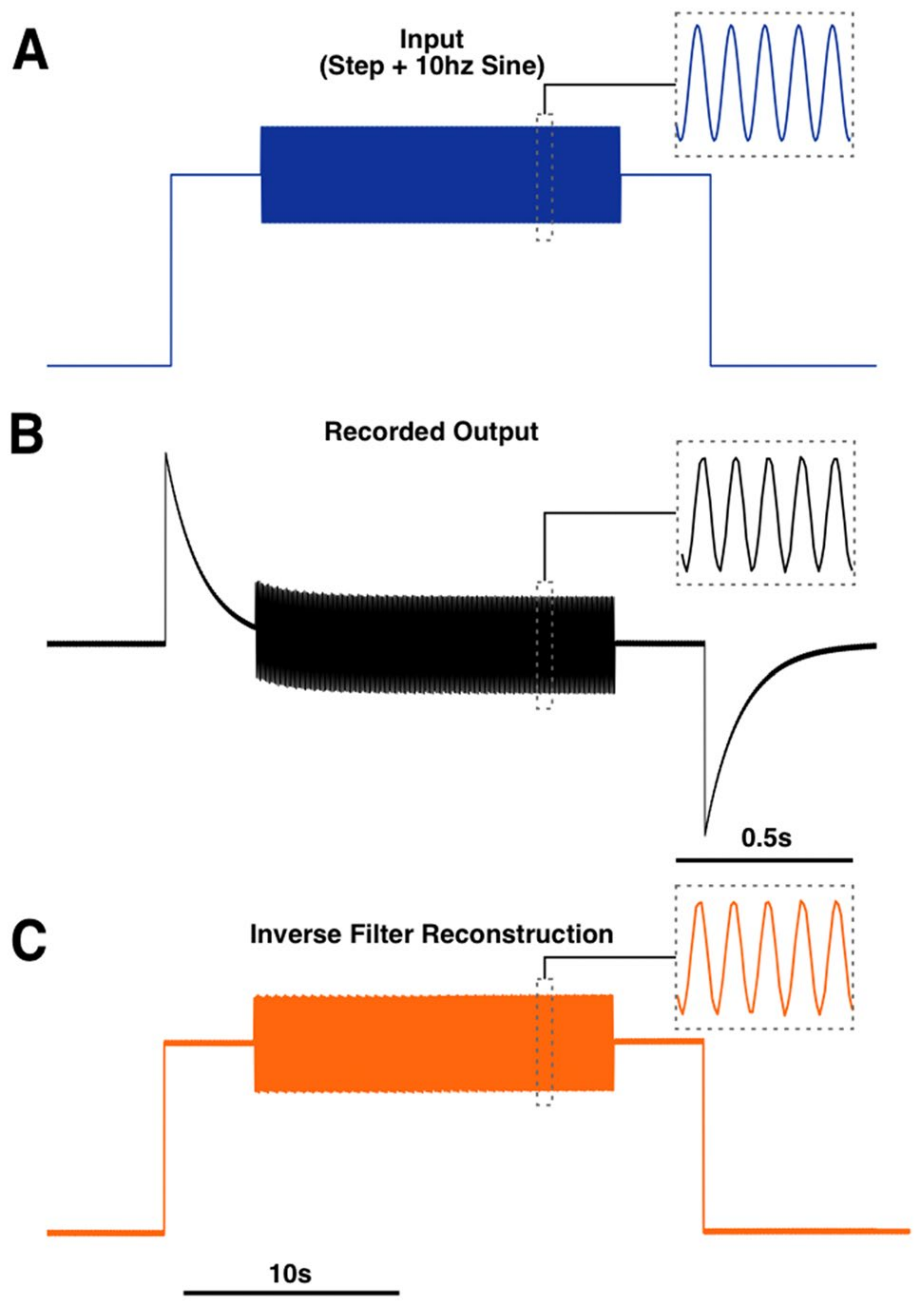
Inverse filter reconstruction of a step function with a 10 Hz oscillation. A signal composed of a 10 Hz oscillation riding on the top of a step function was used as input into the EEG system (**A**). The system’s recorded output (**B**) was deconvolved with the system’s UIR, resulting in a reconstruction of the original step function (**C**). The dotted line insets indicate a 0.5 s window showing the 10 Hz oscillation.

We then tested our inverse filter procedure on two different types of synthetic signals. All inputs were generated digitally in MATLAB and converted to an analog signal using a digital/analog converter to be used as inputs into the EEG system (“Methods”, Supplementary Fig. S2). The first test signal was a step function with a 10 Hz sine overlay (Fig. 4A). As expected, the DC component of the recorded output was greatly attenuated by the system’s 0.1 Hz high-pass filter, while the 10 Hz sine was unaffected (Fig. 4B). Deconvolution of this output with the UIR reconstructed a function that closely resembled the original synthetic input (Fig. 4C).

**Figure 5:**
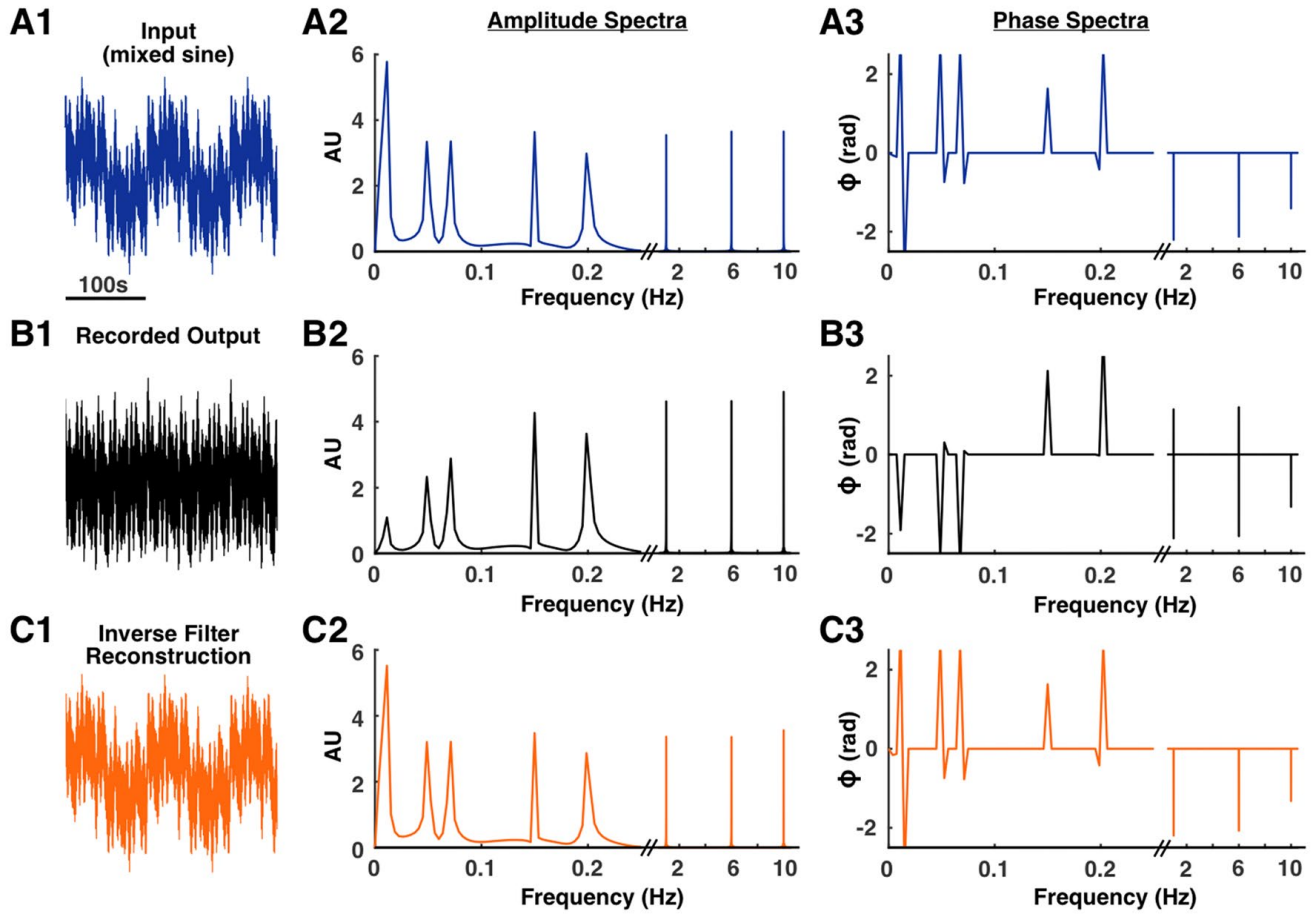
Inverse filter reconstruction of a mixed sine signal. An input signal with known low frequency components was generated by mixing sine waves of varying frequencies (**A1**). The frequency composition was confirmed with an amplitude spectrum (**A2**). As expected, activity in frequencies below 0.1 Hz was attenuated in the recorded output (**B1,B2**). The recording process also introduced a phase distortion (**A3,B3**). Deconvolution of the recorded output with the system’s UIR resulted in a signal with the low frequency components restored (**C1,C2**). This deconvolution process also corrected the phase distortion (**A3,C3**).

Next, we tested an input that was a mixed sine function composed of the following frequencies: 0.01, 0.05, 0.07, 0.15, 0.2, 1, 6, and 10 Hz (Fig. 5A1). Presence of these frequency components were confirmed with an amplitude spectrum (Fig. 5A2). Low frequency components were visibly attenuated in the EEG system’s recorded output, and an amplitude spectrum confirmed this attenuation (Fig. 5B1,B2). Inverse filtering of the recorded output resulted in a mixed sine wave with the low frequency components restored, which was evident by visual inspection of the time series (Fig. 5C1) and confirmed by the amplitude spectra (Fig. 5C2).

The phase spectra of the recorded output showed that the recording process distorts the phase of the input signal (Fig. 5A3,B3). The phase spectra of the original input and reconstruction were similar, demonstrating that the inverse filter was able to correct the phase distortion caused by the EEG system (Fig. 5A3,C3). The fidelity of the reconstruction to the original input was quantified by correlation analysis. The time series, amplitude spectra, and phase spectra of the original input were more highly correlated with the reconstruction than the recorded output (Supplementary Table S1).

### Application of deconvolution-based inverse filter to a known biological signal

**Figure 6:**
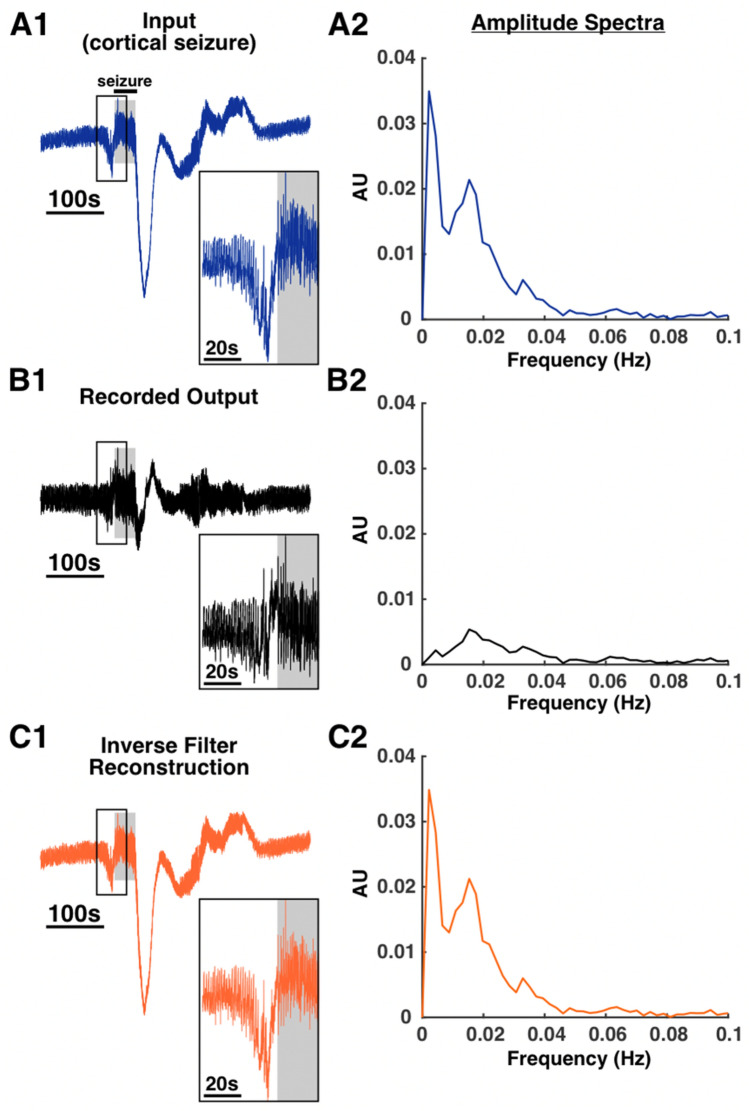
Inverse filter reconstruction of a mouse hippocampal seizure. A recording of a mouse hippocampal seizure with prominent low frequency activity before seizure onset and after seizure offset was used as the input test signal (**A1**,**A2**). This low frequency activity was greatly attenuated in the recorded output (**B1**,**B2**). Deconvolution of this recorded output with the system’s UIR resulted in a reconstruction of the original signal with the low frequency components restored (**C1**,**C2**). Insets in (**A1**), (**B1**), and (**C1**) show a zoomed in view of the pre-seizure low frequency activity. In all panels and insets, the shaded gray background indicates seizure activity.

Next, we wished to test a signal that was of biological relevance. A recording of a hippocampal seizure in a mouse that was available through a public repository was used^14^ (“Methods”). This seizure was originally recorded with a DC amplifier, which allowed preservation and recording of low frequency components. Notably, there is a DC shift prior to the start of the seizure and spreading depolarization activity after the seizure (Fig. 6A1). These features were predictably attenuated in the recorded output (Fig. 6B1). Inverse filtering of this recorded output resulted in a signal that was similar to the original input signal in time, frequency, and phase (Fig. 6C1,C2; Supplementary Fig. S3). This fidelity was confirmed with correlation analysis that showed that for all measures, the input signal was more highly correlated with the reconstruction than the recorded output (Supplementary Table S2).

### Application of deconvolution-based inverse filter to clinical EEG recordings

In the results presented so far, we validated the ability of our inverse filter algorithm to restore low frequencies by using known input signals. Next, we wished to test our inverse filter on a dataset where the ground truth (i.e., the original signal) is unknown. To do this, we used the inverse filter to study low frequency activity in seizure recordings obtained during long-term monitoring of temporal lobe epilepsy patients. Because the EEG set-ups used in the inpatient unit are not portable, characterizing the UIR using the laboratory-based D/A signal generation system is impractical. Therefore, we wished to develop a more convenient method to characterize the UIR. To do this, we took advantage of the fact that the clinical system includes a square wave calibration signal. Because a square wave is simply a series of step functions, we recorded this calibration output, averaged three step responses, and fitted a function to this average to approximate the system’s step response (Supplementary Fig. S4A). To check that the UIR was characterized correctly, this UIR was used to deconvolve the recorded calibration signal, which resulted in a function resembling a square wave (Supplementary Fig. S4B).

**Figure 7:**
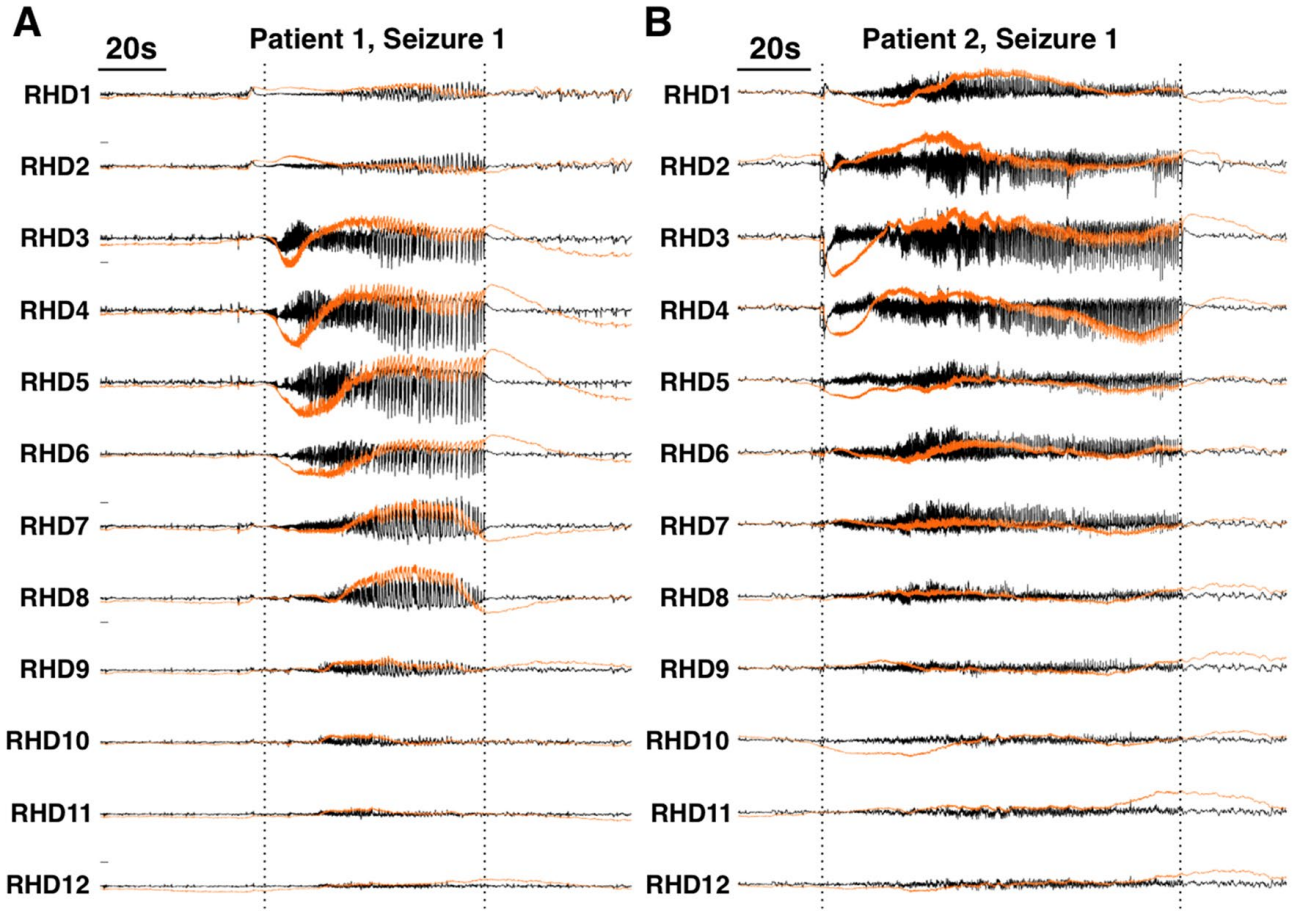
Inverse filter reconstruction of intracranial ictal recordings for two temporal lobe epilepsy patients. Black traces are the raw recordings, and orange traces are the inverse filtered signals. In both patients, large amplitude, low frequency shifts are present at the start of the seizure. *RHD* right hippocampal depth. Vertical dotted lines indicate seizure onsets and offsets.

The UIR derived from the calibration signal was used to deconvolve intracranial recordings obtained from epilepsy patients undergoing presurgical monitoring for medically intractable temporal lobe epilepsy (Supplementary Table S3). Figure 7 shows example reconstructions of right-sided hippocampal depth (RHD) electrodes for two different patients. The depth probe is inserted along the anterior–posterior axis of the hippocampus, with RHD1 being the most anterior electrode (“Methods”).

**Figure 8:**
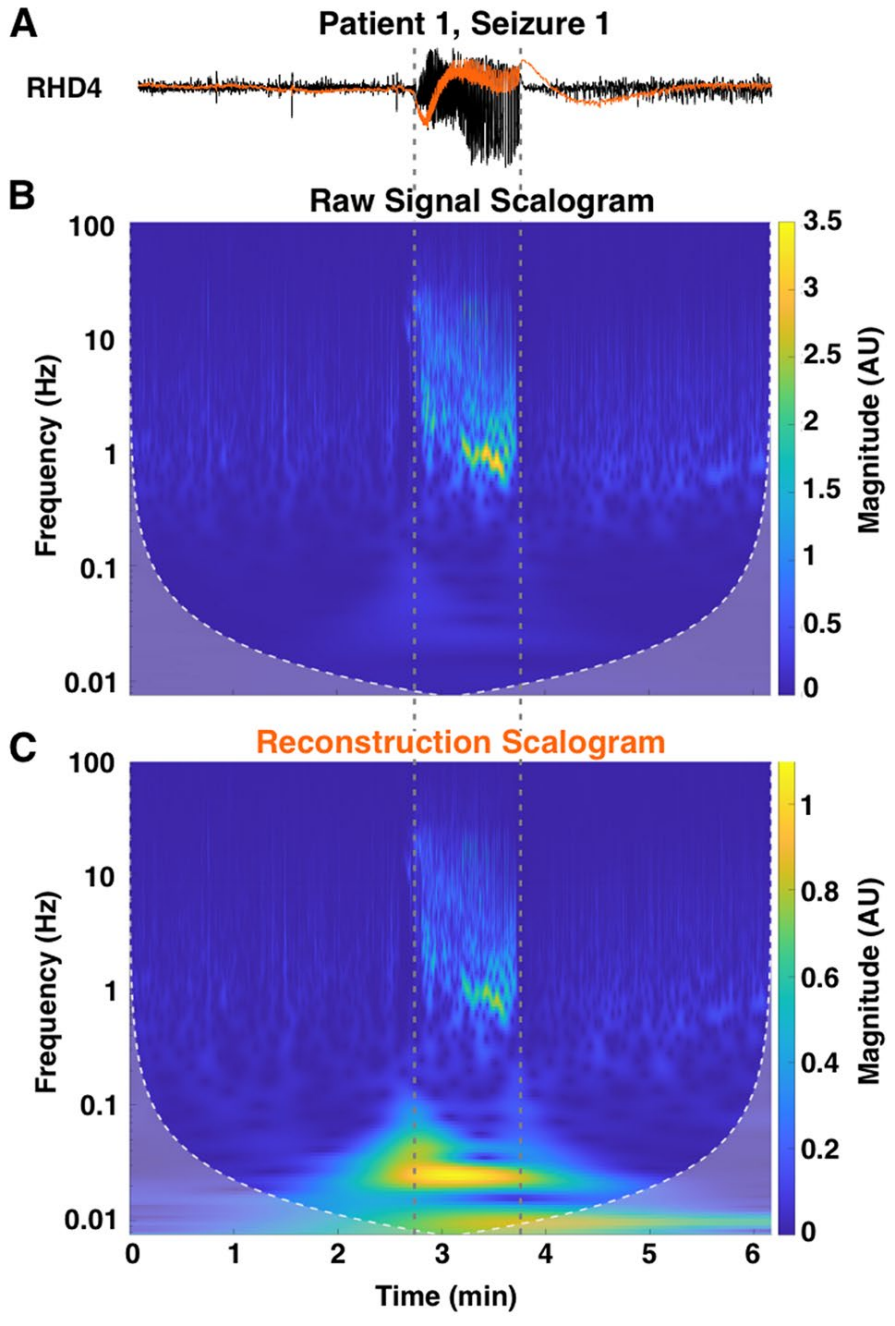
Wavelet analysis of channel RHD4 for the recording for Patient 1, Seizure 1. The inverse filtered reconstruction (orange trace) shows a large low frequency component at seizure onset that is not apparent in the raw recording (black trace) (**A**). Time–frequency analysis confirmed that frequencies below 0.1 Hz are severely attenuated in the raw recording (**B**) but restored in the reconstruction (**C**). The vertical dotted lines indicate seizure onset and offset.

To demonstrate that the reconstructions successfully recovered low frequency signals, high resolution, time–frequency analysis was performed using a Morlet wavelet. An example of the wavelet analysis for a single channel result for Patient 1, Seizure 1 is shown in Fig. 8. A distinct lack of power in frequencies below 0.1 Hz was observed in the analysis of the raw signal (Fig. 8B), as expected by the presence of the 0.1 Hz high-pass filter in the recording system. The inverse filtered reconstruction showed a clear increase in power in the sub-0.1 Hz frequency band that is not present in the raw signal (Fig. 8C). The increase in power was also temporally aligned with the seizure onset (vertical dotted lines).

The restoration of these low frequency components allowed for observations not readily apparent in the raw recordings. In Patient 1, channels RHD3 and RHD4 showed prominent downward shifts at the start of the seizure (Fig. 7A, orange traces). Channel RHD3 also showed a small upward shift prior to seizure onset. These shifts were not meaningfully visible in the raw recordings (Fig. 7A, black traces). Notably, channels RHD3 and RHD4 were identified as the seizure onset zone (SOZ) during the presurgical assessment of this patient. In clinical assessment, the SOZ was determined by visually identifying channels that first showed ictal activity in the conventional clinical band of 1–70 Hz. In the reconstructions, channels RHD5 and RHD6 also showed prominent downward shifts that occurred shortly after the shifts in RHD3 and RHD4. Patient 2 showed similar patterns except the downwards shifts were more concentrated in channels RHD3 and RHD4 (Fig. 7B, orange traces). Although the initial phase of these shifts was visible in the raw recording (Fig. 7B, black traces), their extended time course was only visible in the reconstruction (Fig. 7B, orange traces).

For Patient 2, RHD1-8 were identified as the SOZ during conventional clinical assessment. For both patients, these patterns were replicated in a second seizure recording (Supplementary Fig. S5), showing that this inverse filter method performs reliably and consistently when applied to clinical recordings.

## Discussion

### Advantages of the deconvolution-based inverse filter

One advantage of the deconvolution algorithm presented in this study is its stability. Deconvolution procedures, as with many inverse problems, can be very unstable with results being extremely sensitive to the precision of the unit impulse response (UIR) characterization. We obtain a very precise UIR approximation and increase the stability of our analysis by characterizing the EEG system’s UIR from a measurement of its step response. This continuous approximation then can be differentiated to obtain the system’s UIR (Fig. 2). Curve fitting also allows us to reduce the impact of noise since small fluctuations are smoothed by the continuous approximation of the step response. The stability and precision of our method was demonstrated in our reconstruction of known inputs for both artificial and biologically relevant signals. We also demonstrated that our method is stable when applied to clinical EEG recordings of human seizures.

The deconvolution-based inverse filter presented in this study offers several advantages over current approaches to studying infraslow activity (ISA) in clinical recordings. The use of DC amplifiers has been suggested as a way to preserve low frequency signals in EEG recordings, and some studies have successfully used such set-ups to study ISA^9–12^. This method, however, has not been a practical solution for studying ISA in larger, clinical datasets because DC amplifiers are not used in standard clinical equipment. Furthermore, even DC amplifiers operate within a finite range of amplitudes that is defined by the design and attributes of the amplifier. Because power is required to amplify signals, the amplitude range of amplifiers is bounded by the power supply limit of its electronic components^15^. When signals exceed this range, amplifiers no longer perform linearly, signals become clipped, and useful information is lost (Supplementary Fig. S1).

One distinct advantage of the method presented in this study is the ability to apply this technique retroactively to clinical recordings obtained with an AC amplifier. The method is entirely computational and does not require any specialty equipment. Consequently, the inverse filter algorithm can be applied to any recordings that are obtained from a system for which a unit impulse response (UIR) can be successfully characterized. AC amplifiers (rather than DC amplifiers) are the standard in clinical settings, so the ability to study ISA in recordings obtained from AC amplifiers greatly increases the size of datasets available for analysis. Expanding the number of patient recordings in future analyses will allow the development of more quantitative assessment of ISA.

This method is also able to reconstruct the phase information of the original input signal. Hardware filter components, such as those in EEG systems, introduce phase distortions during the filtering process. Our inverse filter method not only reconstructs the attenuated frequency components but also restores the original phase relationships across the spectrum (Fig. 5A3,B3,C3; Supplementary Fig. S3). The fidelity of the phase spectrum is important as it allows for accurate comparisons of the relative timing of signal components. This timing is particularly important when studying the activity of different frequency bands in relation to the time of seizure onset or when studying relationships such as the coupling between ISA and high frequency oscillations.

Some other studies have posed the idea of an inverse filter to reconstruct low frequency activity in EEG recordings^16,17^. The inverse filter algorithms presented in these studies, however, necessitate characterization of various filter parameters such as resistance and capacitance. This requires knowledge of the precise specifications of the EEG system’s real-time, high-pass filter implementation, which may not be readily available, proprietary, or otherwise difficult to obtain. In contrast, the method presented in this study is completely non-parametric, and no specific attributes of the equipment need to be known to characterize the system’s UIR. This UIR may be obtained for any clinical recording equipment that includes a native calibration signal, which allows this method to be potentially applied more ubiquitously to clinical recordings (Supplementary Fig. S4). Furthermore, the generalizability of this method allows it to be used to study very slow activity in non-clinical datasets since ISA has been observed in physiological contexts^18,19^. Example applications include studies on task-dependent ISA^20^, ISA associated with slow fluctuations in fMRI signals^21^, and sleep-associated ISA^22^.

### Limitations of the deconvolution-based inverse filter

The deconvolution-based inverse filter relies completely on the ability to accurately characterize the UIR of the recording system. Since the UIR is specific to the model of the amplifier, the UIR must be re-characterized if a different model of amplifier is used. Consequently, any updates to hospital recording systems that involve equipment changes will require a recharacterization of the UIR.

This method can be computationally intensive as the computation time scales exponentially with the length of the signal being reconstructed. Depending on the computing resources available, inverse filtering longer signals (> 4 h) may be not practical, although this issue may be bypassed by reconstructing a series of shorter clips and concatenating the results. Significant downsampling of the signals can be also used to reduce computing time since high sampling rates are not necessary to study low frequency activity.

Finally, this method will also reconstruct any low frequency artifacts that can obscure or confound seizure-related ISA. Therefore, any signal being used must be reasonably noise-free, and reconstructions should be screened for results that likely contain artifactual drifts.

### Potential clinical applications

The most distinct advantage of this deconvolution-based inverse filter is that it may be applied retroactively to clinical recordings of seizures. Because ISA has emerged as a topic of interest in identifying epileptogenic tissue (reviewed in Lee et al.^8^), the ability to observe low frequency activity in larger clinical datasets is critical for further investigation. Because conventional clinical EEG equipment utilize AC amplifiers with a high-pass filter, ISA is too severely attenuated in clinical recordings to be visualized and interpreted by neurologists. The inverse filter presented here allows this attenuation to be reversed, rendering the slow drifts to be readily visible and accessible (Fig. 7, Supplementary Fig. S5). While DC amplifiers that may be used in research and clinical settings exist, they are not widely used, limiting the size of datasets that are available for ISA analysis.

Although the exact clinical utility of ISA and its potential mechanistic role in seizure generation is beyond the scope of this study, the clinical examples presented here suggest that distinct patterns of ISA can be observed in the SOZ. For Patient 1, the clinically defined SOZ were channels RHD3 and RHD4. Interestingly, these two channels also showed the most prominent ISA at the seizure onset (Fig. 7A). This is consistent with literature that have suggested that ISA is concordant with the SOZ^23,24^. Large amplitude, downward ISAs were also observed in channels RHD5-6, but they occurred after the ISA in channels RHD3-4 and had a longer time course. These observations only became apparent after the application of the inverse filter. The mechanistic implication of these observations is a target for future studies, but our current results suggest that ISA patterns allow for differentiation between electrodes in a way that is not evident by conventional methods.

For Patient 2, the standard clinical assessment identified a much more diffuse SOZ spanning RHD1 to RHD8. In contrast to this assessment, Patient 2 shows a concentration of ISA power in a smaller range of electrodes, namely electrodes RHD3 and RHD4 (Fig. 7B, orange traces). The concentration and extended time course of this ISA was observable only after application of the inverse filter. These observations are consistent with the literature that have suggested that ISA may allow delineation of a smaller epileptogenic area compared to conventional methods^25–28^. Application of the inverse filter to larger clinical datasets in the future is necessary to investigate the utility of ISA as a marker for seizure generating tissue.

## Methods

### Recording of synthetic and biological test signals

The three synthetic signals (step, step with 10 Hz sine overlay, mixed sine) were generated in MATLAB (MATLAB, Natick, MA, USA) (Figs. 3A, 4A, 5A1). Signals were generated with a sampling rate of 1000 samples/s. The mouse hippocampal seizure recording was downloaded from a publicly available repository (10.5281/zenodo.5655535)^14^ in HDF5 format, downsampled to 1000 samples/s, and converted to a *.mat file (Fig. 6A1). These signals were saved as text files, and a plain-text word processor (WordPad, Microsoft, Windows 10) was used to add a header and convert them into *.atf files that could be read by Clampex (Molecular Devices LLC, San Jose, CA, USA, v.10.4.1.10).

Next, a Clampex episodic stimulation protocol was used with a digital/analog (D/A) converter (Digidata 144A, Molecular Devices LLC, San Jose, CA, USA) to generate an analog output corresponding to each test signal. The output from the D/A converter was attenuated by a factor of 1/1000 using a 10 kOhm/10 Ohm resistor pair in series. This attenuated signal was recorded with a clinical EEG machine (Natus XLTEK Brain monitor with Connex headbox). The recorded signals were exported in the *.edf format for subsequent analysis in MATLAB.

### Unit impulse response curve fitting and deconvolution

Curve fitting to approximate the step response was performed using the *polyfit* interface in MATLAB. A 9th order polynomial was used. Deconvolution was performed using the *deconv* function. Signals were zeroed by subtracting the mean prior to deconvolution. MATLAB scripts that demonstrate this process step-by-step are available in a Github repository (https://github.com/sominlee14/deconvolution_based_inverse_filter).

### Signal processing and statistical analysis

All signal processing and statistical analyses were performed in MATLAB. All reconstructed signals were filtered with a 2nd order high-pass Butterworth filter with a cutoff frequency of 0.005 Hz prior to visualization. This filter step necessary because the deconvolution process in some cases introduces a triangular drift due to the accumulation of rounding errors. The frequency of this drift is entirely dependent on the epoch length of the signal being deconvolved and is equal to $\frac{1}{2 \times epoch \;length}$. To avoid confounding this triangular drift with ictal-associated slow activity, we only used signals with lengths at least three times the period of the lowest frequency of interest. For example, to analyze frequencies around 0.005 Hz, the signal being inverse filtered was at least 600 s long.

### Patients and clinical data acquisition

Clinical EEG recordings were collected from two patients undergoing phase II monitoring for medically intractable right temporal lobe epilepsy at The University of Chicago Adult Epilepsy Center (Supplementary Table S3). Written, informed consent was obtained from patients through a process approved by The University of Chicago Institutional Review Board (IRB). All methods and procedures described in this study were in accordance with the guidelines and regulations approved by The University of Chicago IRB. For both patients, intracranial recordings were collected using depth electrodes placed along right hippocampus. 12-channel depth electrodes were placed along the length of the right hippocampus with contact #1 (RHD1) being most anterior and contact #12 (RHD12) being most posterior. Other intracranial electrodes were placed in other locations, but recordings from these channels were not used for this study.

All patient recordings used in this study were collected using an XLTEK EEG recording system (Natus Neurolink IP EEG Amplifier, INBOX-1166A/B, Natus, Pleasanton, CA, USA). Signals were digitized at 1024 samples/s and referenced to the FCz electrode. The raw broadband (0.1–344 Hz) was converted into *.mat files using a custom C++ routine. Signals were downsampled to 256 samples/s for all subsequent analyses.

## Supplementary Information

Supplementary Information.

## Data Availability

With the exception of patient EEG recordings, all input signals, recordings, and custom scripts used in this study can be found at https://github.com/sominlee14/deconvolution_based_inverse_filter. Patient recordings can be made available upon reasonable request to the corresponding author (W.v.D).
